# Use Pattern of Ophthalmic Antiglaucoma Agents with and without Preservatives: A Cross-Sectional Study

**DOI:** 10.3390/ph16050743

**Published:** 2023-05-12

**Authors:** Luis Fernando Valladales-Restrepo, María Camila Oyuela-Gutiérrez, Ana Camila Delgado-Araujo, Jorge Enrique Machado-Alba

**Affiliations:** 1 Grupo de Investigación en Farmacoepidemiología y Farmacovigilancia, Universidad Tecnológica de Pereira-Audifarma S.A, Pereira 660003, Colombia; 2 Grupo de Investigación Biomedicina, Facultad de Medicina, Fundación Universitaria Autónoma de las Américas, Pereira 660003, Colombia; 3 Semillero de Investigación en Farmacología Geriátrica, Facultad de Medicina, Fundación Universitaria Autónoma de las Américas, Pereira 660003, Colombia

**Keywords:** antiglaucoma agents, latanoprost, preservatives, pharmaceutical, benzalkonium compounds, pharmacoepidemiology, Colombia

## Abstract

Background: Different drugs have been approved to reduce the intraocular pressure. However, most of them contain preservatives to maintain sterility and these can be toxic to the ocular surface. The aim was to determine the patterns of use of antiglaucoma agents and ophthalmic preservatives in a group of patients from Colombia. Methods: A cross-sectional study that identified ophthalmic antiglaucoma agents from a population database of 9.2 million. Sociodemographic and pharmacological variables were considered. Descriptive and bivariate analyses were performed. Results: A total of 38,262 patients were identified, with a mean age of 69.2 ± 13.3 years, and 58.6% were women. A total of 98.8% were prescribed antiglaucoma drugs in multidose containers. The most widely used were prostaglandin analogs (59.9%), especially latanoprost (51.6%) and β-blockers (59.2%). A total of 54.7% of patients received combined management, especially with fixed-dose combination (FDC) drugs (41.3%). A total of 94.1% used antiglaucoma drugs with preservatives (benzalkonium chloride, 68.4%). Conclusions: The pharmacological treatment of glaucoma was very heterogeneous, but the most commonly used therapeutic groups were in accordance with the recommendations of clinical practice guidelines but with differences by sex and age. Most of the patients were exposed to preservatives, especially benzalkonium chloride, but the wide use of FDC drugs can minimize toxicity on the ocular surface.

## 1. Introduction

Glaucoma is a chronic progressive optic neuropathy characterized by damage to the optic nerve and the nerve fibers of the retina leading to loss of peripheral vision and occasionally of central vision [1,2,3]. It is classified according to the anatomy of the anterior chamber angle (open: normal iridocorneal angle without iris occlusion; and closed: narrow iridocorneal angle with iris occlusion) or according to the rapidity of appearance (acute or chronic) and the etiology (primary/idiopathic or secondary) [2,4]. The global prevalence of glaucoma is 3.5% in people between 40 and 80 years of age, with open-angle glaucoma being the most common condition (3.1%), while closed-angle glaucoma is less frequent (0.5%) [5].

Glaucoma is one of the leading causes of blindness in the world [1,2,4]. The progression of this pathology is attenuated by decreasing intraocular pressure, which is achieved with topical medications and procedures such as laser trabeculoplasty and incisional surgery [1,4,6,7,8]. First-line therapy is usually pharmacological, while the most invasive procedures are used in patients with an inadequate response to medications [4]. Some medications increase the output of aqueous humor from the eye (prostaglandin analogs -PGA-, α2-adrenergic agonists -AA-, and muscarinic agonists -MA-), and others decrease its production (AA, β-blockers -BB- and carbonic anhydrase inhibitors -CAI-), leading to a reduction in intraocular pressure [1,3,4,6,7,8]. According to clinical practice guidelines, the initial pharmacological management should include PGAs and, as an alternative, BB. If these options are not tolerated or the objective of reducing intraocular pressure is not achieved, other therapeutic groups should be used, such as CAI, AA, or MA, or a combination of medications [6,7,8].

Preservatives are a requirement of eye drops in multidose containers to maintain sterility and avoid bacterial contamination [9,10]. Most preservatives act relatively nonspecifically as detergents or by oxidative mechanisms and therefore cause secondary effects on the ocular surface and periorbital structures [9,11,12]. Benzalkonium chloride (BAK) is the most widely used preservative in ophthalmology and is more toxic than other newer preservatives, such as Polyquad, Purite, and SofZia [9,11,12]. Ophthalmic preservatives, especially BAK, have been identified as implicated in the development of ocular surface disease [12,13]; its prevalence is between 49–59% [12], and this condition negatively impacts adherence to the antiglaucoma treatment regimen and the patient’s quality of life [12,13]. Topical medication without preservatives could be recommended mainly for patients with ocular surface disease, severe or refractory glaucoma, a proven allergy to preservatives, and contact lenses, among others [10,12].

Knowledge of prescription patterns as evidence from the real world helps to assess the appropriate use of medications and the degree of adherence to the recommendations of clinical practice guidelines [14]. Its inappropriate use can lead to less effectiveness, greater adverse reactions, and increased costs [14]. Similarly, knowing the type of ophthalmic preservatives would provide information on the potential safety risk to which patients are exposed [12,13]. However, the information on the patterns of use of antiglaucoma drugs in the country is limited (BB and PGA predominate) [15], and the data available on the preservatives present in them are unknown. Internationally, there are few studies that address this topic, but it has been shown that most patients use antiglaucoma drugs with preservatives (84.0–96.0%); however, these reports do not characterize the type of preservative [16,17,18,19]. Another limitation of these studies is the low number of patients included [16,17,18,19].

The Colombian health system offers universal coverage to the entire population through two affiliation regimes: the contributory regime that is paid by workers and employers; and the subsidized regime that is responsible for the insurance of all people without the ability to pay, which has a benefit plan that includes a heterogeneous group of medications for the treatment of glaucoma. The objective of the study was to determine the patterns of use of antiglaucoma agents and ophthalmic preservatives present in these drugs in a group of patients affiliated with the Colombian Health System in 2022.

## 2. Results

A total of 38,262 patients in 187 cities were identified as taking some ophthalmic antiglaucoma medication. A total of 58.6% (*n* = 15,346) were women, and the average age was 69.2 ± 13.3 years. A small percentage of 0.5% (*n* = 186) were under 18 years old, 2.4% (*n* = 923) were 18–39, 28.7% (*n* = 10,964) were 40–64, and 68.4% (*n* = 26,189) were 65 or older. According to the geographic regions, the patients were mainly found in the Caribbean region (*n* = 12,971; 33.9%), followed by the Bogotá-Cundinamarca region (*n* = 10,428; 27.3%), Pacific region (*n* = 6767; 17.7%), Central region (*n* = 6567; 17.2%), and Eastern Amazonia–Orinoquía region (*n* = 1529; 4.0%). A total of 68.6% (*n* = 26,231) were insured by the health system’s contributory scheme, and 31.4% (*n* = 12,031) were insured by the subsidized scheme.

### 2.1. Type of Glaucoma and Comorbidities

A total of 59.7% (*n* = 22,824) had a diagnosis of unspecified glaucoma, 35.3% (*n* = 13,494) open-angle glaucoma, and 5.1% (*n* = 1944) closed-angle glaucoma, in which blindness was reported in 1.2% (*n* = 465) of all cases. A total of 71.0% (*n* = 27,183) of the patients had some chronic pathology, the most frequent being arterial hypertension (AH) (*n* = 19,971; 52.2%), diabetes mellitus (DM) (*n* = 9585; 25, 1%), and hypothyroidism (*n* = 6625; 17.3%), with a predominance in women (Table 1) and in those aged 65 or older (Table 2). A total of 19.7% (*n* = 7553) presented some ophthalmic comorbidity, such as cataracts (*n* = 2868; 7.5%), conjunctivitis (*n* = 1531; 4.0%), and dry eye (*n* = 1485; 3.9%).

### 2.2. Antiglaucoma Use Pattern

The vast majority of patients were prescribed antiglaucoma drugs in multidose containers (*n* = 38,175; 99.8%). The most widely used therapeutic groups were PGAs (*n* = 22,907; 59.9%) and BBs (*n* = 22,635; 59.2%) (Table 1 and Table 2), and the most prescribed drug was latanoprost (*n* = 19,747; 51.6%) (Table 3). Most of the patients took a combination of medications (*n* = 20,923; 54.7%), especially with FDC drugs (*n* = 15,796; 41.3%) (Table 1 and Table 2). Twenty-seven different management schemes were found, the most common being monotherapy with a PGA (*n* = 10,436; 27.3%), followed by quadruple therapy with PGA + BB + AA + CAI (*n* = 4909; 12.8%) and triple therapy with BB + AA + CAI (*n* = 4334; 11.3%) (Table 1 and Table 2). Antiglaucoma drugs were prescribed mainly by general medicine (*n* = 29,575; 77.3%) and ophthalmology (*n* = 3023; 7.9%). Table 1 and Table 2 show differences in drug use patterns according to sex and age group, and Table 3 shows the prescription patterns, frequency of use, distribution by sex, age, pharmaceutical form, and presence or not of ophthalmic preservatives.

### 2.3. Comedications

A total of 73.4% (*n* = 28,090) of the patients received systemic comedications, predominantly antihypertensive and diuretic (*n* = 19,585; 51.2%), lipid-lowering (*n* = 15,371; 40.2%), and analgesic or anti-inflammatory (*n* = 10,403; 27.2%). Similarly, 36.5% (*n* = 13,981) had some ophthalmic comedication, especially ocular lubricants (*n* = 12,778; 33.4%), corticosteroids (*n* = 1988; 5.2%), and antibiotics (*n* = 647; 1.7%) (Table 1 and Table 2).

### 2.4. Ophthalmic Preservatives

Forty-nine different trade names for antiglaucoma medications were found, and the type of preservative could be determined in 85.7% of them. In seven products, the information on the type of preservative was not recorded on the label or in the technical data sheet of the drug. Thus, the majority of patients, 94.1% (*n* = 36,001), received antiglaucoma agents with preservatives. BAK predominated (*n* = 26,161; 68.4%), followed by sodium perborate (*n* = 492; 1.3%) and Polyquad (*n* = 135; 0.4%) (Table 1, Table 2 and Table 3). In 37.8% (*n* = 14,466), the type of preservative was unknown. A total of 88.7% (*n* = 33,934) of the patients received only antiglaucoma agents with preservatives, 5.9% (*n* = 2261) received only antiglaucoma agents free of preservatives, and 5.4% (*n* = 2067) received both antiglaucoma agents with preservatives and preservative-free.

## 3. Discussion

This study made it possible to characterize the prescription pattern of ophthalmic antiglaucoma drugs and the preservatives present in them as evidence of drug use in the real world in a group of patients affiliated with the Colombian Health System. These findings can be useful for health care, academic, and scientific personnel in making decisions regarding the risks faced by their patients. Further, these findings can contribute to strengthening the practices of the appropriate use of medications among physicians as a way to reduce problems related to their use in the country.

The average age of the patients in this study was similar to that found in other publications (67.1–72.0 years) [15,16,17,20,21]. However, it contrasts with some reports from Asian countries where the age was lower (55.0–61.3 years) [22,23]. Most of the patients were women, which is consistent with what is reported in the literature (54.2–72.9%) [15,17,20,21,24,25]. In this study, it was found that arterial hypertension and diabetes mellitus were the most common pathologies, which is consistent with other investigations [26,27,28]. High blood pressure and diabetes mellitus can contribute to the progression of glaucoma [29,30]. Cataracts were the most common ophthalmic comorbidity, as identified by Hwang et al. in Korea [26]. This pathology can induce pupillary blockage and occlusion of the iridocorneal angle, giving rise to closed-angle glaucoma, so its management is crucial in these patients [31].

PGAs were the most prescribed therapeutic group, especially in elderly individuals, which is consistent with other studies (48.0–58.8%) [16,20,25,32,33], but higher than that found in Taiwan and China (22.8% and 30.2%, respectively) [22,24]. Similarly, the most prescribed drug was latanoprost, as evidenced in other studies (28.0–50.0%) [20,24,34]. BB were also used with high frequency in this study, which was higher than that reported in the literature (9.4–44.1%) [16,22,32,33]. However, in general, the pattern of use of antiglaucoma agents is in line with the recommendations of the clinical practice guidelines, which suggest PGA as the first line of treatment or, failing that, BB [6,7,8].

Combination therapy with antiglaucoma agents predominated in this report, contrasting with other pharmacoepidemiological investigations where monotherapy prevailed (54.0–78.4%) [20,22,32,33]. This is probably due to the methodological differences used in the studies (e.g., type of study, way of identifying the cases, characteristics of the patients) [20,22,32,33], as our group of patients may have had a greater severity of the pathology. In this sense, men and increasing age have been associated with a greater risk of blindness and vision loss [31]. In this group of patients, therapy with various antiglaucoma agents predominated. Management with several medications requires that patients have multiple applications per day and poses difficulties in adherence and efficacy, as well as in safety due to greater exposure to preservatives [3,12]. However, in this report, the majority used FDC drugs, similar to findings described by Yan et al. in China (36.6%) [23] and very different from what was found in other studies (3.7–21.3%) [22,33]. The use of these drugs reduces the total amount of drops and preservatives applied per day, saves costs, improves tolerability and compliance, and prevents the washing effect that results from the sequential application of multiple drops [3,35].

The use of antiglaucoma agents with preservatives predominated widely in this report. The information available from studies with real-world evidence addressing this topic is limited [16,17,18,19]. However, in Germany, Wolfram et al. identified that 96.0% of patients with glaucoma used antiglaucoma agents with preservatives [18]. In France, Chamard et al. documented that 84.6% of patients with glaucoma were exposed to some preservative [16]. In Spain, Pérez-Bartolomé et al. described that 84.4% of glaucoma patients were exposed to preservatives [19]. Similarly, in Belgium, France, Italy, and Portugal, Jaenen et al. described that 84.0% of patients received antiglaucoma agents with preservatives [17]. In neither of the four investigations was the type of preservative characterized [16,17,18,19]. In a study carried out in Tunisia, 80.0% of the patients received antiglaucoma drugs with BAK [36]. In this report, a wide use of BAK was found, which is the most commonly used preservative in ophthalmology [9,11,21] and is linked to cytotoxic damage to the epithelial cells of the conjunctiva and cornea, which can lead to signs and symptoms of ocular surface disease [9,11] and contributes to the adherence and persistence of the use of antiglaucoma agents [10]. There are some strategies available to minimize exposure to BAK, such as (1) using alternative preservatives such as Polyquad, Purite, or SofZia (used in less than 2% of medications in this report), (2) using antiglaucoma agents without preservatives (used in little more than one-tenth of medications), (3) using FDC drugs in those who need to be managed with multiple drugs (used in three-quarters of medications in combination treatment), and (4) using drugs with the greatest efficacy (PGAs were used in more than half of the medications) [9,12].

It is striking that in some antiglaucoma patients, the type of preservative used was unknown. The regulations of the Food and Drug Administration (FDA) establish several elements that must be present in the information about medicines, such as active and inactive ingredients [37]. However, the local regulations in charge of the National Institute for Food and Drug Surveillance (INVIMA) are not very specific in this specific aspect [38]. This could explain the lack of data for some brands of antiglaucoma medications. Drug labeling is the primary tool for communicating essential information about the safe and effective use of a pharmaceutical product [39]. It is important that information on ophthalmic preservatives is available to medical personnel and the general population, so potential adverse reactions are known and can be prevented in patients with a history of hypersensitivity to the preservative and those who wear contact lenses [9,11,21].

Some limitations are recognized in the interpretation of the results, since access to medical records was not obtained to verify the clinical characteristics of the patients, such as the etiology of glaucoma, chronicity, severity, and complications, as well as the efficacy of the treatment and possible adverse drug reactions. Information from complementary studies such as tonometry, pachymetry, perimetry, gonioscopy, and ophthalmoscopy was not available. Similarly, the drugs prescribed outside the health system or not delivered by the dispensing company that the patients may have received are unknown. However, this study has a very important number of cases, distributed in most of the national territory, involving both the contributory and subsidized regimes of the country’s health system.

## 4. Materials and Methods

### 4.1. Study Design and Patients

An observational cross-sectional study was carried out on the prescription patterns of antiglaucoma agents as well as the identification of their preservatives based on a database of approximately 9.2 million people affiliated with the Colombian Health System. The individuals were served by four health insurance companies, corresponding to approximately 25.3% of the active affiliated population of the contributory or payment scheme and 13.1% of the state-subsidized scheme, which comprise 18.8% of the Colombian population. The medication dispensing database contains sociodemographic variables (age, sex, dispensing city, and affiliation regime), pharmacological (medication, pharmaceutical shape, dose, and prescriber doctor), and main and secondary diagnoses [40,41]. This database is the most widely used source of secondary information for studies with evidence in the real world in Colombia, which allows for research on the use and safety of medications [41]. To date, more than 200 investigations have been carried out and published [40].

The identification of the patients was made from the dispensing of ophthalmic antiglaucoma drugs (BB, PGA, AA, CAI, and MA) from 1–31 October 2022. The drugs included were all those that were approved and that are marketed in the country. The first date of dispensing of the antiglaucoma was considered the patient’s index date. Inclusion criteria: patients of any age, sex and origin were selected and received medical consultations as outpatients. Exclusion criteria: None.

### 4.2. Variables

Based on the information about the drug consumption of the affiliated population, which was systematically obtained from the dispensing company (Audifarma S.A., Colombia), a database was designed that allowed the following groups of patient variables to be collected:

1. Sociodemographic: sex, age (<65 years and ≥65 years), health system affiliation regime (contributory or subsidized), and place of origin. The place of origin was categorized by departments according to the regions of Colombia, taking into account the classification of the National Administrative Department of Statistics (DANE), as follows: Bogotá-Cundinamarca region, Caribbean region, Central region, Eastern region, Pacific region, and Amazon–Orinoquía region;
2. Clinical: The clinics identified the type of glaucoma and comorbidities in patients selected no more than 90 days from their index dates, using the codes of the International Classification of Diseases, version 10 (ICD-10). The type of glaucoma was categorized into closed-angle glaucoma (H402), open-angle glaucoma (H401), and unspecified glaucoma (H403-H406, H408, H409, H420, and H428);
3. Pharmacological:
  - Type of prescriber: general practitioner, ophthalmologist, others;
  - Antiglaucoma drugs: BB (betaxolol, timolol, levabunolol), PGA (bimatoprost, latanoprost, tafluprost, travoprost, isopropyl unoprostone), AA (apraclonidine, brimonidine), CAI (brinzolamide, dorzolamide), and MA (pilocarpine) in pharmaceutical form (single-dose or multidose container and fixed-dose combination (FDC));
  - Ophthalmic preservatives: the presence or absence of ophthalmic preservatives was identified from the label and technical sheet of each antiglaucoma agent (Benzalkonium chloride (BAK); Polyquartenium 1 (Polyquad); Sodium perborate; Stabilized Oxychloro Complex (SOC, Purite); Borate, sorbitol, propylene glycol, and zinc (SofZia);
  - Comedications: drugs prescribed in the 30 days following the index date were identified and grouped into the following categories: systemic comedications (antidiabetic, antihypertensive and diuretic, thyroid hormone, antiulcer, antidepressant and anxiolytic, analgesic and anti-inflammatory, antiepileptic, lipid-lowering, among others) and ophthalmic comedications (ocular lubricants, antibiotics, corticosteroids, antihistamines, nonsteroidal anti-inflammatory drugs, and sympathomimetics, among others).

### 4.3. Ethical Statement

The protocol was approved by the Bioethics Committee of the Technological University of Pereira in the category of research without risk (Endorsement code: 03-091120). The ethical principles established by the Declaration of Helsinki were respected.

### 4.4. Data Analysis

The data were analyzed with the statistical package SPSS Statistics, version 26.0, for Windows (IBM, USA). Descriptive analysis was performed with frequencies and proportions for the qualitative variables and measures of central tendency and dispersion for the quantitative variables by means and standard deviation. The comparison of quantitative variables was performed using the Mann‒Whitney U test and X^2^ or Fisher’s exact test for categorical variables. A level of statistical significance was established at *p* < 0.05.

## 5. Conclusions

With these findings, we can conclude that the pharmacological treatment was very heterogeneous, but the most commonly used therapeutic groups are in accordance with the recommendations of the clinical practice guidelines, albeit with differences by sex and age. Most of the patients were prescribed antiglaucoma drugs in multidose and were exposed to preservatives, especially benzalkonium chloride, that could contribute to the development of ocular surface disease, but the wide use of FDC drugs can minimize toxicity on the ocular surface.

## Figures and Tables

**Table 1:** Comparison of some sociodemographic, comorbidity, and pharmacological variables by sex in patients who received antiglaucoma therapy in Colombia.

Variables	Women		Men		*p*
	*n* = 22,413	%	*n* = 15,849	%	
Age, mean ± SD	69.4 ± 13.1		68.7 ± 13.4		<0.001
≥65 years	15,346	68.5	10,843	68.4	0.910
Glaucoma type	-	-	-	-	-
Unspecified glaucoma	13,384	59.7	9440	59.6	0.763
Open angle glaucoma	7644	34.1	5850	36.9	<0.001
Angle closure glaucoma	1385	6.2	559	3.5	<0.001
Comorbidities	-	-	-	-	-
Arterial hypertension	12,185	54.4	7786	49.1	<0.001
Diabetes mellitus	5704	25.4	3881	24.5	0.032
Hypothyroidism	4868	21.7	1757	11.1	<0.001
Benign prostatic hyperplasia	0	0.0	2246	14.2	<0.001
Dyslipidemia	1099	4.9	641	4.0	<0.001
Therapeutic groups (antiglaucomatous)	-	-	-	-	-
PGA	13,390	59.7	9517	60.0	0.548
BB	12,576	56.1	10,059	63.5	<0.001
AA	8157	36.4	7440	46.9	<0.001
CAI	7983	35.6	6870	43.3	<0.001
MA	325	1.5	45	0.3	<0.001
Type of treatment	-	-	-	-	-
Monotherapy	11,043	49.3	6296	39.7	<0.001
Combined	11,370	50.7	9553	60.3	
Fixed-dose combination drug	8399	37.5	7397	46.7	<0.001
Treatment schemes	-	-	-	-	-
PGA	6641	29.6	3795	23.9	<0.001
PGA + BB + AA + CAI	2397	10.7	2512	15.8	<0.001
BB + AA + CAI	2217	9.9	2117	13.4	<0.001
BB	2430	10.8	1404	8.9	<0.001
PGA + BB	1759	7.8	1132	7.1	0.010
BB + CAI	1613	7.2	1049	6.6	0.029
AA	1374	6.1	912	5.8	0.126
PGA + BB + CAI	923	4.1	701	4.4	0.145
PGA + AA	690	3.1	562	3.5	0.011
BB + AA	671	3.0	563	3.6	0.002
Use of ophthalmic preservatives	-	-	-	-	-
Benzalkonium chloride	14,885	66.4	11,276	71.1	<0.001
Unknown preservative	8762	38.1	5704	36.0	<0.001
Free of preservatives	2440	10.9	1888	11.9	0.002
Other preservatives	390	1.7	236	1.5	0.057
Systemic medications	-	-	-	-	-
Antihypertensives and diuretics	11,931	53.2	7654	48.3	<0.001
Lipid-lowering	9411	42.0	5960	37.6	<0.001
Analgesics and anti-inflammatories	7210	32.2	3193	20.1	<0.001
Ulcerative	6518	29.1	3172	20.0	<0.001
Antidiabetics	4764	21.3	3296	20.8	0.278
Ophthalmic medications	-	-	-	-	-
Eye lubricants	7624	34.0	5154	32.5	0.002
Corticosteroids	1229	5.5	759	4.8	0.003
Antibiotics	397	1.8	250	1.6	0.147
Sympathomimetics	289	1.3	201	1.3	0.856
Antihistamines	282	1.3	188	1.2	0.529

SD: Standard deviation; PGA: Prostaglandin analogs; BB: beta blockers; AA: alpha-adrenergic agonists; CAI: carbonic anhydrase inhibitors; MA: muscarinic agonists.

**Table 2:** Comparison of some sociodemographic, comorbidity, and pharmacological variables by age in patients who received antiglaucoma therapy in Colombia.

Variables	≥65 Years		<65 Years		*p*
	*n* = 26,189	%	*n* = 12,073	%	
Women	15,346	58.6	7067	58.5	0.910
Glaucoma type	-	-	-	-	-
Unspecified glaucoma	15,197	58.0	7627	63.2	<0.001
Open angle glaucoma	9609	36.7	3885	32.2	<0.001
Angle closure glaucoma	1383	5.3	561	4.6	0.009
Comorbidities	-	-	-	-	-
Arterial hypertension	15,604	59.6	4367	36.2	<0.001
Diabetes mellitus	7445	28.4	2140	17.7	<0.001
Hypothyroidism	5329	20.3	1296	10.7	<0.001
Benign prostatic hyperplasia	1882	7.2	364	3.0	<0.001
Dyslipidemia	1087	4.2	653	5.4	<0.001
Therapeutic groups (antiglaucomatous)	-	-	-	-	-
PGA	16,316	62.3	6591	54.6	<0.001
BB	15,466	59.1	7169	59.4	0.548
AA	10,508	40.1	5089	42.2	<0.001
CAI	10,285	39.3	4568	37.8	0.007
MA	167	0.6	203	1.7	<0.001
Type of treatment	-	-	-	-	-
Monotherapy	11,576	44.2	5763	47.7	<0.001
Combined	14,613	55.8	6310	52.3	
Fixed-dose combination drug	10,838	41.4	4958	41.1	0.558
Treatment schemes	-	-	-	-	-
PGA	7260	27.7	3176	26.3	0.004
PGA + BB + AA + CAI	3466	13.2	1443	12.0	<0.001
BB + AA + CAI	2801	10.7	1533	12.7	<0.001
BB	2516	9.6	1318	10.9	<0.001
PGA + BB	2103	8.0	788	6.5	<0.001
BB + CAI	1787	6.8	875	7.2	0.130
AA	1327	5.1	959	7.9	<0.001
PGA + BB + CAI	1181	4.5	443	3.7	<0.001
PGA + AA	936	3.6	316	2.6	<0.001
BB + AA	801	3.1	433	3.6	0.007
Use of ophthalmic preservatives	-	-	-	-	-
Benzalkonium chloride	18,054	68.9	8107	67.1	<0.001
Unknown preservative	9957	38.0	4509	37.3	0.208
Free of preservatives	2967	11.3	1361	11.3	0.872
Other preservatives	445	1.7	181	1.5	0.152
Systemic medications	-	-	-	-	-
Antihypertensives and diuretics	15,825	60.4	3760	31.1	<0.001
Lipid-lowering	12,215	46.6	3156	25.1	<0.001
Analgesics and anti-inflammatories	7929	30.3	2474	20.5	<0.001
Ulcerative	7720	29.5	1970	16.3	<0.001
Antidiabetics	6306	24.1	1754	14.5	<0.001
Ophthalmic medications	-	-	-	-	-
Eye lubricants	9201	35.1	3577	29.6	<0.001
Corticosteroids	1467	5.6	521	4.3	<0.001
Antibiotics	477	1.8	170	1.4	0.004
Sympathomimetics	371	1.4	119	1.0	<0.001
Antihistamines	352	1.3	118	1.0	0.002

PGA: Prostaglandin analogs; BB: beta blockers; AA: alpha-adrenergic agonists; CAI: carbonic anhydrase inhibitors; MA: muscarinic agonists.

**Table 3:** Prescription patterns, frequency of use, distribution by sex, age, pharmaceutical form (multidose container), and presence or absence of ophthalmic preservatives in 38,262 outpatients with antiglaucoma dispensations in Colombia.

Antiglaucomatous	*n* = 38,262	%	Sex		Age	Pharmaceutical Form	Preservatives	
			F (%)	M (%)	Mean (SD)	Multidose (%)	Yes (%)	No (%)
Latanoprost	19,747	51.6	59.2	40.8	70.0 (12.4)	100.0	96.4	3.7
Dorzolamide/Timolol/Brimonidine	8976	23.5	49.9	50.1	69.0 (13.3)	100.0	72.3	28.4
Timolol	7338	19.2	60.6	39.4	69.1 (13.3)	100.0	100.0	0.0
Brimonidine	5123	13.4	56.0	44.0	68.1 (13.5)	100.0	100.0	0.0
Dorzolamide/Timolol	4114	10.8	58.6	41.4	69.0 (14.1)	100.0	78.9	22.5
Bimatoprost	1660	4.3	51.8	48.2	69.2 (13.0)	99.8	99.6	0.4
dorzolamide	1182	3.1	64.3	35.7	72.8 (14.1)	100.0	100.0	0.0
Brimonidine/Timolol	1154	3.0	53.3	46.7	68.4 (13.1)	100.0	100.0	0.0
Latanoprost/Timolol	412	1.1	59.2	40.8	71.2 (11.9)	100.0	100.0	0.0
Travoprost	401	1.0	51.9	48.1	70.4 (13.3)	100.0	99.3	0.7
Brinzolamide/Timolol	379	1.0	59.4	40.6	69.9 (15.0)	100.0	100.0	0.0
Pilocarpine	370	1.0	87.8	12.2	62.6 (12.9)	100.0	100.0	0.0
Brimonidine/Brinzolamide	314	0.8	52.5	47.5	76.2 (12.0)	100.0	100.0	0.0
Bimatoprost/Timolol	270	0.7	51.5	48.5	71.4 (16.0)	97.8	97.0	0.3
Bimatoprost/Timolol/Brimonidine	242	0.6	52.5	47.5	71.4 (16.0)	100.0	100.0	0.0
Tafluprost	155	0.4	66.5	33.5	72.2 (13.9)	50.3	0.0	100.0
Travoprost/Timolol	143	0.4	61.5	38.5	67.4 (15.4)	100.0	94.4	5.6

F: Female; M: Male; SD: Standard deviation.

## Data Availability

protocols.io.

## References

[1] Stein J.D., Khawaja A.P., Weizer J.S. (2021). Glaucoma in Adults-Screening, Diagnosis, and Management: A Review. JAMA.

[2] Jonas J.B., Aung T., Bourne R.R., Bron A.M., Ritch R., Panda-Jonas S. (2017). Glaucoma. Lancet.

[3] Wang T., Cao L., Jiang Q., Zhang T. (2021). Topical Medication Therapy for Glaucoma and Ocular Hypertension. Front. Pharmacol..

[4] Wagner I.V., Stewart M.W., Dorairaj S.K. (2022). Updates on the Diagnosis and Management of Glaucoma. Mayo Clin. Proc. Innov. Qual. Outcomes.

[5] Tham Y.C., Li X., Wong T.Y., Quigley H.A., Aung T., Cheng C.Y. (2014). Global prevalence of glaucoma and projections of glaucoma burden through 2040: A systematic review and meta-analysis. Ophthalmology.

[6] European Glaucoma Society Terminology and Guidelines for Glaucoma (2017). Chapter 3: Treatment principles and options Supported by the EGS Foundation: Part 1: Foreword; Introduction; Glossary. In European Glaucoma Society Terminology and Guidelines for Glaucoma, 4th Ed. Br. J. Ophthalmol..

[7] National Institute for Health and Care Excellence Glaucoma: Diagnosis and Management. NICE Guideline [NG81]. 26 January 2022. https://www.nice.org.uk/guidance/ng81.

[8] Gedde S.J., Vinod K., Wright M.M., Muir K.W., Lind J.T., Chen P.P., Li T., Mansberger S.L. (2021). American Academy of Ophthalmology Preferred Practice Pattern Glaucoma Panel. Primary Open-Angle Glaucoma Preferred Practice Pattern^®^. Ophthalmology.

[9] Goldstein M.H., Silva F.Q., Blender N., Tran T., Vantipalli S. (2022). Ocular benzalkonium chloride exposure: Problems and solutions. Eye.

[10] Thygesen J. (2018). Glaucoma therapy: Preservative-free for all?. Clin. Ophthalmol..

[11] Fineide F., Lagali N., Adil M.Y., Arita R., Kolko M., Vehof J., Utheim T.P. (2022). Topical glaucoma medications—Clinical implications for the ocular surface. Ocul. Surf..

[12] Andole S., Senthil S. (2023). Ocular Surface Disease and Anti-Glaucoma Medications: Various Features, Diagnosis, and Management Guidelines. Semin. Ophthalmol..

[13] Li G., Akpek E.K., Ahmad S. (2022). Glaucoma and Ocular Surface Disease: More than Meets the Eye. Clin. Ophthalmol..

[14] Jain S., Jain P., Moghe V.V., Seth V., Upadhyaya P., Abhijit K., Goyal J. (2015). A systematic review of prescription pattern monitoring studies and their effectiveness in promoting rational use of medicines. Perspect. Clin. Res..

[15] Valladales-Restrepo L.F., Machado-Alba J.E. (2020). Potentially inappropriate prescriptions of anticholinergic medications in patients with closed-angle glaucoma. Int. Ophthalmol..

[16] Chamard C., Larrieu S., Baudouin C., Bron A., Villain M., Daien V. (2020). PSS10 Preservative-free versus preserved glaucoma eye drops and occurrence of glaucoma surgery. A retrospective cohort study based on the French national health insurance information system, 2008–2016. Acta Ophthalmol..

[17] Jaenen N., Baudouin C., Pouliquen P., Manni G., Figueiredo A., Zeyen T. (2007). Ocular Symptoms and Signs with Preserved and Preservative-Free Glaucoma Medications. Eur. J. Ophthalmol..

[18] Wolfram C., Stahlberg E., Pfeiffer N. (2019). Patient-Reported Nonadherence with Glaucoma Therapy. J. Ocul. Pharmacol. Ther..

[19] Pérez-Bartolomé F., Martinez-De-La-Casa J.M., Arriola-Villalobos P., Pérez C.F., Polo V., Feijoo J.G. (2017). Ocular Surface Disease in Patients under Topical Treatment for Glaucoma. J. Glaucoma.

[20] Sousa D.C., Leal I., Nascimento N., Neves C.M., Tuulonen A., Pinto L.A. (2017). Use of Ocular Hypotensive Medications in Portugal: PEM Study: A Cross-Sectional Nationwide Analysis. Eur. J. Gastroenterol. Hepatol..

[21] Ramli N., Supramaniam G., Samsudin A., Juana A., Zahari M., Choo M.M. (2015). Ocular Surface Disease in Glaucoma: Effect of Polypharmacy and Preservatives. Optom. Vis. Sci..

[22] Lin J.C. (2015). The use of ocular hypotensive drugs for glaucoma treatment: Changing trend in Taiwan from 1997 to 2007. J. Glaucoma.

[23] Yan J., Xiong X., Shen J., Huang T. (2022). The use of fixed dose drug combinations for glaucoma in clinical settings: A retrospective, observational, single-centre study. Int. Ophthalmol..

[24] Yu L., Ding K., Luo L., Yu Z. (2020). Prescribing trends of glaucoma drugs in six major cities of China from 2013 to 2017. PLoS ONE.

[25] Fujita A., Hashimoto Y., Matsui H., Yasunaga H., Aihara M. (2022). Recent Trends in Treatment and Associated Costs of Primary Angle-Closure Glaucoma: A Retrospective Cohort Study. Ophthalmol. Glaucoma.

[26] Hwang I.C., Suh H.S., Choi S. (2022). Factors Associated with Non-Adherence to Glaucoma Treatment in a Korean Nationwide Survey. Iran. J. Public Health.

[27] Wändell P., Carlsson A.C., Ljunggren G. (2021). Systemic diseases and their association with open-angle glaucoma in the population of Stockholm. Int. Ophthalmol..

[28] Lee S.H., Kim G.A., Lee W., Bae H.W., Seong G.J., Kim C.Y. (2017). Vascular and metabolic comorbidities in open-angle glaucoma with low- and high-teen intraocular pressure: A cross-sectional study from South Korea. Acta Ophthalmol..

[29] Leeman M., Kestelyn P. (2019). Glaucoma and Blood Pressure. Hypertension.

[30] Pinto L.A., Ferreira J.T., Costa L., Cunha J.P., Amado D. (2015). Diabetes Mellitus as a Risk Factor in Glaucoma’s Physiopathology and Surgical Survival Time: A Literature Review. J. Curr. Glaucoma Pract..

[31] Sun Y., Chen A., Zou M., Zhang Y., Jin L., Li Y., Zheng D., Jin G., Congdon N. (2022). Time trends, associations and prevalence of blindness and vision loss due to glaucoma: An analysis of observational data from the Global Burden of Disease Study 2017. BMJ Open.

[32] Pimenta G., Sousa D.C., Leal I., Marques-Neves C., Abegão Pinto L. (2019). Prescription pattern of ocular hypotensive drugs in Por-tugal and its comparison with the European guidelines—PEM Study. Acta Ophthalmol..

[33] Schwartz G.F., Patel A., Naik R., Lunacsek O., Ogbonnaya A., Campbell J. (2021). Characteristics and Treatment Patterns of Newly Diagnosed Open-Angle Glaucoma Patients in the United States: An Administrative Database Analysis. Ophthalmol. Glaucoma.

[34] Choi S., Choi J.A., Kwon J.W., Park S.M., Jee D. (2018). Patterns of care for glaucoma patients in Korea from 2002 to 2013 using the national health insurance service claims data. Medicine.

[35] Konstas A.G., Schmetterer L., Costa V.P., Holló G., Katsanos A., Denis P., Quaranta L., Irkec M., Castejón M.A., Teus M.A. (2020). Current and emerging fixed combination therapies in glaucoma: A safety and tolerability review. Expert Opin. Drug Saf..

[36] Lajmi H., Hmaied W., Achour B.B., Zahaf A. (2021). Risk Factors for Ocular Surface Disease in Tunisian Users of Preserved An-tiglaucomatous Eye Drops. J. Curr. Ophthalmol..

[37] Guidance, Compliance, & Regulatory Information FDA’s Labeling Resources for Human Prescription Drugs. https://www.fda.gov/drugs/laws-acts-and-rules/fdas-labeling-resources-human-prescription-drugs.

[38] Ministerio de Salud de Colombia Decreto 677 de 1995: Régimen de Registros y Licencias, El Control de Calidad, Así Como El Régimen de Vigilancia Sanitaria de Medicamentos, Cosméticos, Preparaciones Farmacéuticas a Base de Recursos Naturales, Productos de Aseo, Higiene y Limpieza y Otros Productos de Uso Doméstico y se Dictan Otras Disposiciones Sobre la Materia. https://www.funcionpublica.gov.co/eva/gestornormativo/norma.php?i=9751.

[39] Clarridge K.E., Chin S.J., Stone K.D. (2022). Overview of FDA Drug Approval and Labeling. J. Allergy Clin. Immunol. Pract..

[40] Fármaco Online Audifarma, SA. https://www.audifarma.com.co/farmaco-online/.

[41] Franco J.S., Vizcaya D. (2020). Availability of secondary healthcare data for conducting pharmacoepidemiology studies in Colombia: A systematic review. Pharmacol. Res. Perspect..

